# Psychological features in male and female adolescents with eating disorders: is it the same condition?

**DOI:** 10.1007/s40519-023-01583-y

**Published:** 2023-06-28

**Authors:** Anna Riva, Giulia Purpura, Simona Di Guardo, Mariella Falbo, Maria Pigni, Renata Nacinovich

**Affiliations:** 1 School of Medicine and Surgery, University of Milano Bicocca, 20900 Monza, Italy; 2 Child and Adolescent Health Department, Fondazione IRCCS San Gerardo dei Tintori, 20900 Monza, Italy

**Keywords:** Eating disorders, Adolescent male, Gender, Anorexia nervosa, Bulimia

## Abstract

**Purpose:**

Eating disorders (EDs) are psychiatric disorders with a typical prevalence in adolescence. EDs have long been wrongly considered female gender-bound disorders, resulting in a systematic underrepresentation of males in EDs research. The main goal of the present study is exploring the clinical and psychological characteristics of adolescent males with EDs in comparison with females.

**Methods:**

In this observational and retrospective study, 14 males and 28 females hospitalized for eating disorders during the adolescent age (from 12 to 17.11 years) were recruited. Main clinical data (age, BMI, duration of illness), behavioural characteristic of the disorder (over-exercising, self-harm, purging-behaviours) and psychological symptoms (Eating Disorders Inventory-3rd edition—EDI-3, Symptom Checklist-90-Revised—SCL-90, Children’s Global Assessment Scale—C-GAS) were collected and examined for significant correlations with severity of body mass index (BMI).

**Results:**

Adolescent males show a peculiar and more severe psychopathological profiles partially influenced by BMI and characterized by purging-behaviours, over-exercising, obsessive–compulsive behaviour, anxiety, and psychoticism.

**Conclusion:**

This study suggests a gender-specific profile of adolescent males with EDs, which may be considered in diagnosis and treatment.

***Level III*:**

Evidence obtained from retrospective well-designed case–control study.

## Introduction

Eating disorders (EDs) represent one of the most prevalent psychiatric disorders in adolescence [1] and are characterized by irregular eating attitudes and behaviours ranging from excessive weight concern and body shape dissatisfaction to extreme weight control methods and binge eating [2]. EDs are associated with severe medical and psychiatric morbidity and high rates of mortality [3, 4] and, among all the mental health disorders, are the most gendered with a typical presentation in females [5].

Historically, EDs have long been wrongly considered female gender-bound disorders and male presentation as unusual, therefore resulting in a systematic underrepresentation of males in EDs research [6]. Reasons of this underestimation seem to be many, both attributable to DSM-IV diagnostic criteria (mostly tailored for the female gender) [7] and for the use of female-oriented tools, and also due to the fact that adolescent and young adult males could be less likely to seek treatment than females as victims of a double stigma: the stigma of suffering of a psychiatric disorder and an additional stigma (shame, discrimination, etc.) of suffering from a female-specific disorder [8, 9].

In the recent years instead, different aspects contributed to improve the interest and the research in this field. Firstly, the introduction of DSM-5 [3, 10], with the removal of amenorrhea criterion, and a more flexible evaluation of significantly low body weight [7], that allowed to include in the EDs an increased number of male-gendered subjects. Secondary, a significant improvement in the diagnostic definition of EDs in males has been characterized by the introduction in DSM-5 of the category of avoidant/restrictive food intake disorder (ARFID), a disorder that typically affect more frequently males and younger than other EDs [11]. Finally, some recent literature has contributed to clarify the epidemiology of EDs in males [12] with emerging evidence that indicates an increasing rate of EDs in males faster than in females [13, 14].

Concerning clinical characteristics, adolescent and young adult males with EDs generally report less weight concern, drive to thinness, and body dissatisfaction than females [15, 16]. As a matter of fact, body dissatisfaction in adolescent and young adult males with EDs seems to be centred around being “bigger” and more muscular [13, 17, 18] differently from females where is usually associated with a desire to be thinner. Other controversial gendered differences may include a minor self-induced vomiting than females [19–21] and over-exercising, seen that being an athletes may be a risk factor for adolescent boys compared to females [22, 23]. Homosexuality has also been identified as specific risk factor for the development of EDs in this population [13] and people who identify as trans, gender non-binary or gender diverse are at two to four times greater risk of EDs than their cisgender counterparts [24].

Moreover, studies on psychiatric comorbidity of EDs in males showed that males with EDs, particularly those affected by binge-eating disorder and bulimia nervosa, are more likely to report a greater array of psychiatric comorbidities [25, 26] as anxiety, psychosis spectrum disorders, narcissistic and antisocial personality disorders [27], impulse-control problems [28], as well as obsessive–compulsive disorder and drug and alcohol abuse [5]. Finally, not less important, a severity of the disorder in males added to a scarce knowledge of EDs in males contributes to an increase of length of hospitalization in comparison to females [29] and a longer illness-duration pre-hospitalization [30].

Despite the interesting results of these recent research, anyway the knowledge regarding the expression of EDs in males remains scarce and results contrasting, especially in paediatric and adolescent age, also because the prevalence of studies have been conducted on adult population. A recent study conducted by our research group [31] on a large sample of 287 children and adolescents, 27 males and 260 females with EDs, in order to identify similarities and differences, outlined differences especially correlated with age, with an average age of males lower than females (median age 12.65 vs. 15.145). Further results showed a different distribution of typology of EDs in middle childhood and middle adolescents, with the totality of males presenting a diagnosis of ARFID in middle childhood, while in middle adolescents, the 70% of males a diagnosis of restricting-type anorexia nervosa and the 30% of eating disorder not otherwise specified (EDNOS), differently from females where the distribution was more heterogeneous. Finally, results indicated that the psychological profile relating to the eating disorder was similar in early and middle adolescence, but worst and major levels of general psychological distress emerged in early adolescent males than females.

Based on these premises, the present study aims to improve the understanding of the clinical and psychological characteristics of male adolescents with EDs through:

- the comparison of the clinical and psychological profiles between adolescent males and females with EDs,
- the study of the correlation between BMI and psychological profiles in the group of males and females separately.

For the first objective, it is hypothesized that male adolescents with EDs show a more general, and less specific on eating disorders, psychopathology than females. For the second objective, the hypothesis is that the psychological profile in males is more independent by BMI than in females.

## Methods

### Sampling and data collection

We performed an observational and retrospective study on a sample of 42 adolescents hospitalized at Child and Adolescent Mental Health Department of “Fondazione IRCCS San Gerardo dei Tintori” and University of Milano-Bicocca (Monza, Italy) from 2013 to 2021. As first, we selected from medical records the male subjects according to these inclusion criteria: (i) age between 12 and 17 years and 11 months; (ii) hospitalization for severe malnutrition due to eating disorders, diagnosed according to DSM-5 criteria. Successively, females matched for age and subtype of eating disorder were selected with a proportion of 2:1 (females:males). For both groups, exclusion criteria were: (i) severe intellectual disability, (ii) severe psychosis, (iii) alcohol or drug addictions, (iv) any somatic or neurological comorbidities with a strong influence on life activities.

According to this procedure, 14 males and 28 females hospitalized for eating disorders during the adolescent age were recruited. The study was conducted in accordance with the Declaration of Helsinki and approved by the Ethics Committee of ASST Monza (protocol code: PXNPI; date of approval: 22 October 2020) for studies involving humans. Written informed consent has been obtained from the patients to publish this paper. Data were collected from subjects’ medical records and included:

- anthropometric and clinical data upon medical evaluation in hospital (age, BMI calculated as kg/m^2^, duration of disease, subtype of eating disorder);
- information related to behavioural characteristic of disorders (in particular the presence or the absence of symptoms as over-exercising, self-harm, purging-behaviours);
- clinical information about the psychological profiles through the collection of scores obtained through 3 clinical questionnaires (see Measures Section).

### Measures

Three questionnaires, commonly used in clinical practice, were administered during hospitalization and the score and sub-scores of all recruited patients were collected from subjects’ medical records:

- EDI-3 (Eating Disorders Inventory-3rd edition): a 91-item self-reporting questionnaire intended to provide a psychological profile of symptoms related to eating disorders and a quantitative measure of their presence and intensity (for patients from 13 to 53 years). The Italian version of the EDI-3 [32] provides 12 primary single scales (Drive for Thinness -DT-, Bulimia -B-, Body Dissatisfaction -BD-, Low Self-Esteem -LSE-, Personal Alienation -PA-, Interpersonal Insecurity -II-, Interpersonal Alienation -IA-, Interoceptive Deficits -ID-, Emotional Dysregulation -ED-, Perfectionism -P-, Asceticism -A-, and Maturity Fears -MF-) and six composite scales (Eating Disorder Risk Composite -EDRC-, Ineffectiveness Composite -IC, Interpersonal Problems Composite -IPC, Affective Problems Composite -APC-, Overcontrol Composite -OC-, and General Psychological Maladjustment Composite -GPMC-). For each scale, higher scores reflect worse symptomatology [33]. The reliability coefficients of the scales range from 0.80 and 0.90, and test–retest reliability coefficients for the various composite scales are between 0.93 and 0.98.
- SCL-90R (Symptom Checklist-90-Revised): a self-report questionnaire designed to assess psychological problems and psychopathological symptoms on adolescents and adults. This original measure, and the validated Italian version [34], consists of 90 items rated on a five-point Likert scale that assesses nine symptom dimensions [somatization (SOM), obsessive–compulsive (OC), interpersonal sensitivity (INT), depression (DEPR), anxiety (ANX), hostility (HOS), phobic anxiety (PHOB), paranoid ideation (PAR), psychoticism (PSY)]. The SCL90-R test also provides 3 indexes: General Symptomatic Index (GSI), which discriminates subjects at high risk of psychiatric disorder and in a psychopathological condition; Positive Symptom Total (PST), which corresponds to the number of symptoms checked; Positive Symptom Distress Index (PSDI), a ratio between the sum of all items and the PST. For each index, scores between 55 and 65 are considered borderline, higher than 65 pathological. The Italian version, used in this research, show good internal coherence for all subscales (α values between 0.70 and 0.96) [34].
- C-GAS (Children’s Global Assessment Scale): a scale for the assessment of the global social adjustment of the patients. The Italian version has been used in previous samples of adolescents with psychiatric morbidities. The scale is separated into 10-point sections that are headed with a description of the level of global functioning and followed by examples matching the given interval. The final score ranges from 1 (the most impaired level of global functioning) to 100 (the superior level of global functioning). The authors report an inter-rater reliability of 0.84, and a test–retest reliability of 0.85 [35].

### Statistical analyses

All analyses were carried out using JASP 0.16.1 software. A p-value below or equal to 0.05 was interpreted as significant. Shapiro–Wilk test for normality was performed. Since that the sample size was limited and that, within the two groups, that are variables with normal and not-normal distribution, we decided to use a non-parametric approach.

Descriptive analyses were reported where appropriate: the continuous variables were expressed through median, mean ± standard deviation of the corresponding distribution; the categorical variables were expressed as absolute or percentage frequencies.

To compare the two groups (Group of Males and Group of Females) about psychological/psychiatric outcomes at EDI-3, C-GAS and SCL-90 and socio-demographic data, the Mann–Whitney test for two independent populations was performed. Chi-squared test was performed to compare categorical variables (for example 0 for the absence or 1 for the presence of over-exercising, self-harm, and purging-behaviours).

Finally, in the two groups separately, a two-tail bivariate non-parametric correlation test (Spearman test) between BMI and psychological/psychiatric outcomes (EDI-3, C-GAS and SCL-90) was also carried out.

## Results

### Differences between the two groups

As reported in Table 1, the two groups were very similar and homogeneous in term of age and BMI, so they are well confrontable. Considering the entire sample, 39 subjects (26 females:13 males) have a diagnosis of anorexia nervosa, and 3 subjects (2 females:1 male) have a diagnosis of eating disorder not otherwise specified. Illness duration prior to the first evaluation was slightly longer in males than in females (*p* = 0.039). A higher frequency of over-exercising (*p* = 0.020) and purging-behaviours (*p* = 0.003) emerged in males than females.

**Table 1:** Clinical data of the two groups

	Females	Males	*p*-value
Age (ys.ms)			
Median	14.6	14.7	0.641
Mean	14.9	14.8	
Std. deviation	1.6	1.6	
Range	12.6–17.6	12.5–17.4	
Education (%)			
Lower secondary school	28.6	35.7	0.637
Higher secondary school	71.4	64.3	
BMI			
Median	15.4	15.5	0.915
Mean	15.9	15.9	
Std. deviation	2.4	2.5	
Range	11.9–21.6	12.6–22.0	
Duration of disease (ms)			
Median	6	9.5	0.039*
Mean	8.9	12.5	
Std. deviation	13.4	11.9	
Range	2–75	3–50	
Over-exercising (%)			
Yes	32.1	71.4	0.020*
No	67.9	28.6	
Self-harm (%)			
Yes	10.7	21.4	0.350
No	82.9	78.6	
Purging-behaviours (%)			
Yes	0	28.6	0.003***
No	100	71.4	

Absolute values and statistical differences by Mann–Whitney tests between the two groups for age, BMI, and duration of disease were reported; percentages frequencies and statistical differences by Chi-square tests between the two groups were reported for education and for over-exercising, self-harm and purging-behaviours symptoms. The asterisks indicate a significant difference between conditions: *p ≤ 0.05, **p ≤ 0.01, ***p ≤ 0.005

**Fig. 1:**
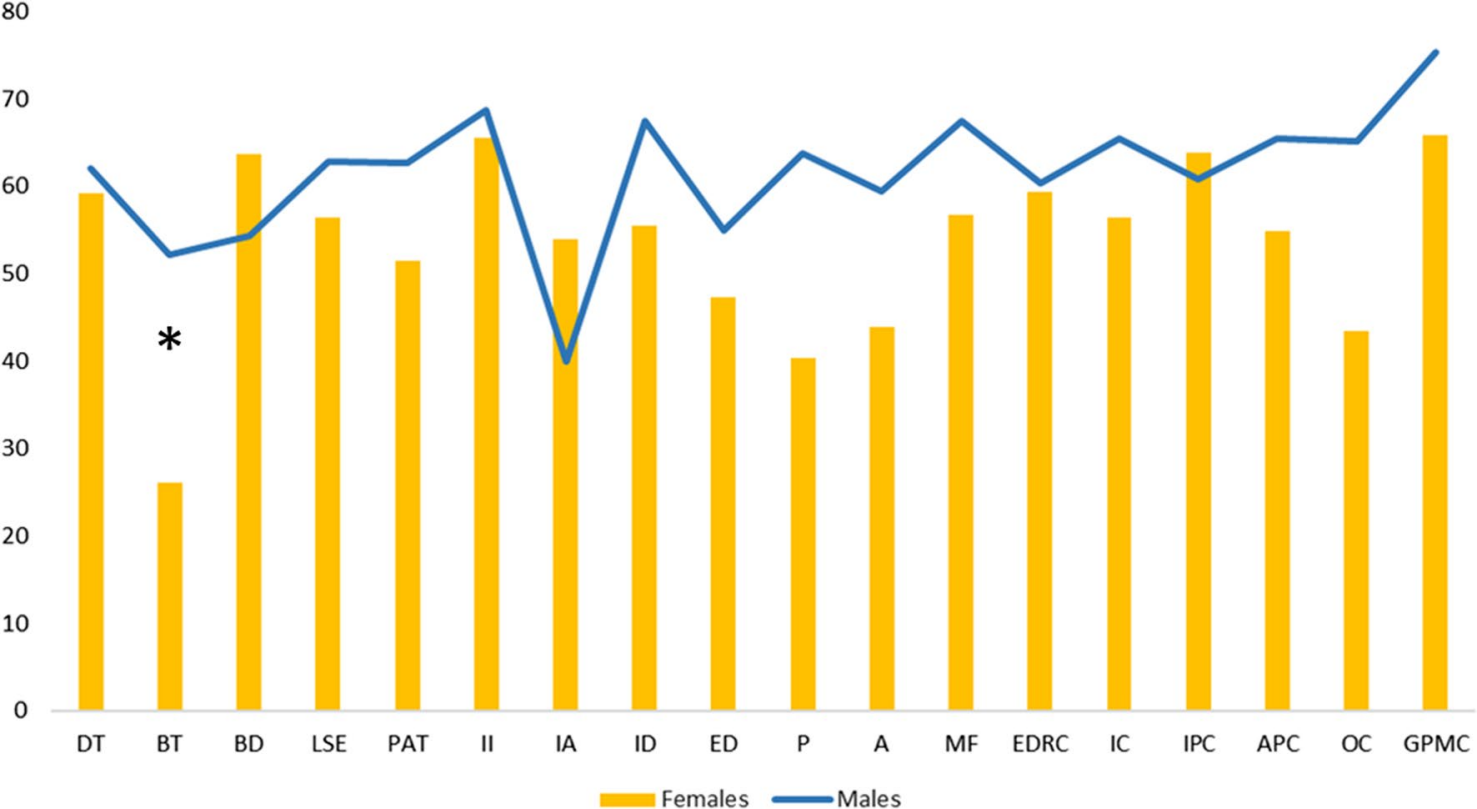
Graphic representation of means of scores at subscales of EDI-3. The asterisks indicate a significant difference between conditions: *p ≤ 0.05, **p ≤ 0.01, ***p ≤ 0.005 (Drive for Thinness -DT-, Bulimia -B-, Body Dissatisfaction -BD-, Low Self-Esteem -LSE-, Personal Alienation -PA-, Interpersonal Insecurity -II-, Interpersonal Alienation -IA-, Interoceptive Deficits -ID-, Emotional Dysregulation -ED-, Perfectionism -P-, Asceticism -A-, and Maturity Fears -MF-, Eating Disorder Risk Composite -EDRC-, Ineffectiveness Composite -IC, Interpersonal Problems Composite -IPC, Affective Problems Composite -APC-, Overcontrol Composite -OC-, and General Psychological Maladjustment Composite -GPMC-)

**Fig. 2:**
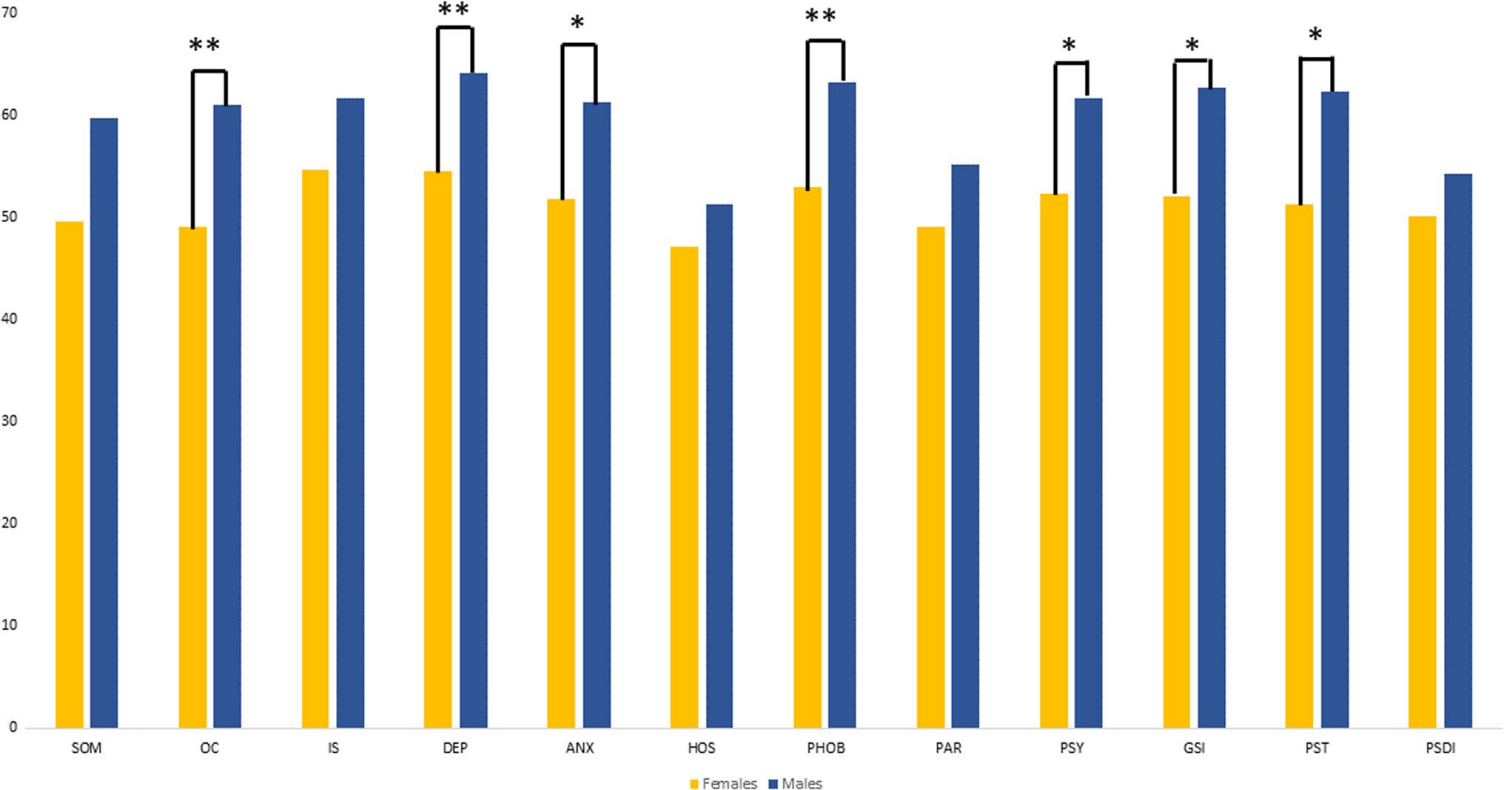
Graphic representation of means of scores at subscales of SCL90-R. The asterisks indicate a significant difference between conditions: *p ≤ 0.05, **p ≤ 0.01, ***p ≤ 0.005 (somatization -SOM-, obsessive–compulsive -OC-, interpersonal sensitivity -INT-, depression DEPR-, anxiety -ANX-, hostility -HOS-, phobic anxiety -PHOB-, paranoid ideation -PAR-, psychoticism -PSY-, General Symptomatic Index -GSI-, Positive Symptom Total -PST-, Positive Symptom Distress Index -PSDI-)

The Mann–Whitney test showed significant statistical differences in EDI-3 (see Fig. 1 and Table 2) only for the Bulimia Scale (*p* = 0.037) and a weak difference in Perfectionism Scale (*p* = 0.056), in which males obtained higher scores than females, while no difference was evident at the C-GAS scores (see Table 2). Moreover, at the SCL-90R, males obtained higher scores than females in the all domains and global indices, even if statistical differences were evident in obsessive–compulsive behaviour (*p* = 0.012), in depression (*p* = 0.016), in anxiety (*p* = 0.034) and phobic anxiety (*p* = 0.013), in psychoticism (*p* = 0.023), in the GSI, that measures overall psychological distress (*p* = 0.23) and in PST, that reports number of self-reported symptoms (*p* = 0.021) (see Fig. 2 and Table 2).

**Table 2:** Main scores in psychopathological traits by psychological/psychiatric measures (Scores of Composite Scales at EDI-3, Total Score of C-GAS, Scores of Main Indexes at SCL90-R)

		Females mean (SD)	Males mean (SD)	*p*-value
EDI-3	EDCR	59.4 (29.6)	60.4 (29.4)	0.942
	IC	56.4 (31.8)	65.4 (28.1)	0.487
	IPC	63.8 (31.7)	60.8 (24)	0.418
	APC	54.8 (32.3)	65.4 (30.1)	0.287
	OC	43.4 (34.1)	65.1 (28.4)	0.078
	GPMC	65.9 (29.8)	75.4 (27.8)	0.534
C-GAS	TOTAL	56.35 (11.3)	57 (14.9)	0.821
SCL-90R	GSI	52.1 (13.7)	62.8 (12.9)	0.023*
	PST	51.5 (14.2)	62.4 (13.8)	0.021*
	PSDI	50.2 (12.5)	54.3 (10.3)	0.153

*p*-value at Mann–Whitney tests were reported

The asterisks indicate a significant difference between conditions: *p ≤ 0.05, **p ≤ 0.01, ***p ≤ 0.005

### Correlations within the two groups

Considering only the group of males, significant negative correlations were found between the BMI and interoceptive deficits scale (*ρ* − 0.625; *p* = 0.030), while two weak negative correlations emerged between BMI and the emotional dysregulation scale (*ρ* − 0.560; *p* = 0.058) and between BMI and the overcontrol composite scale (*ρ* − 0.570; *p* = 0.053). According to these results, there was an association between the BMI decrease and the increase of the scores in these 3 domains. No correlations were found between BMI and C-GAS and between BMI and SCL-90R scores.

With regard to the group of females, surprising significant positive correlations were found between BMI and drive for thinness scale (*ρ* 0.428; *p* = 0.023) and a weak positive correlation between BMI and Eating Disorder Risk Composite scale (*ρ* 0.374; *p* = 0.054). According to these results, there was an association between the increase of BMI and the increase of psychological symptomatology specifically linked to the eating symptomatology. No correlation was found between BMI and C-GAS score and between BMI and SCL-90R scores.

## Discussion

In this study, the clinical and psychological characteristics of male adolescents with EDs were investigated in the comparison with a sample of female adolescents.

The first result of this study concerns that the clinical characteristics in term of diagnosis and BMI between the two groups are comparable, thus making the two groups homogeneous for the further analyses on the psychological profile. The only difference emerges in illness-duration before hospitalization, longer in males than females. This data is in line with literature of a longer illness-duration in males’ pre-hospitalization [30], probably due both to the scarcity of knowledge on this phenomenon and to the greater difficulty to access to mental health services for adolescent males for the double stigma previously described.

Starting from the first hypothesis of our study, results confirm that male adolescents with EDs show a more severe and general psychopathology than females. As a matter of fact, as regards the psychological characteristics related to the eating disorder, the adolescent males show more serious behaviours at bulimia scale and a weak significance in perfectionism scale. Typically, the tendency to self-oriented perfectionism is often at the heart of relentless efforts at weight control and it was frequently described in males associated with bulimia and with a wide presence of bingeing and purging behaviour aimed to develop heavy musculature [36]. Research report that bulimic behaviours are more common among boys and young men with body image concerns related to muscularity, through a phenomenon referred to as a “cheat meal”, in which they show the tendency to consume many calories and prohibited foods as a periodic deviation from what is customarily a muscularity-oriented dietary practice [37]. This aspect is indirectly confirmed in our study by the greater presence in males not only of purging-behaviours but also of over-exercising, and it is in line with data on literature previously described, concerning the differences in compensatory behaviours (less laxative use and more vomiting) [19] and a body dissatisfaction centred to muscularity [12, 13] and not to a desire to be thinner.

The main difference in the psychological profile has been observed in the SCL90-R profiles, a scale that evaluates the psychological problems and psychopathological symptoms. A greater general impairment emerges in males in different scales. In particular, males show higher and significant scores in the Obsessive–Compulsive scale (that measures on thoughts, impulses and actions that are experienced as irresistible and unremitting and that are of an ego-alien or unwanted nature), in the Depression scale (that reflects the representative range of the manifestations of clinical depression, included dysphoric mood and affect, signs of withdrawal of life interest, lack of motivation and loss of vital energy), in the Phobic Anxiety scale (that assesses the presence of disproportionate responses related to persistent fears for a person, place, object or situation, capable of provoking avoidance or escape reactions), and in the Psychoticism scale (that evaluates the presence of schizoid manifestations and the emergency of early symptoms of schizophrenia). As previously described, to the best of our knowledge, results on research about gender differences in psychopathologies of adolescents with EDs are still scarce and contrasting [31, 38–40] and anyway seem to indicate that the presence of psychopathological symptoms other than eating disorders influence the course of the illness and the possibility of a recovery. Bean and colleagues [41] further affirm that females may present more psychopathology symptoms at the start of treatment but make better progress than males in reducing these symptoms over time. Weltzin and colleagues [42] otherwise indicate that the comorbidity of obsessive–compulsive behaviours in eating disordered of males and females influences the longer length of stay and increases the severity of eating disorder symptoms. Finally, differences in psychoticism confirm literature data that report psychotic symptoms in males with EDs in a higher percentage compared to females [43–45].

Proceeding with the second hypothesis that the psychological profile in males is more independent by the severity of BMI than in females, results only partially confirm our statement. As a matter of fact, results show a relation between the BMI and the psychopathological profiles, differently from the initial hypothesis, but the interesting aspect is that a more severity of BMI influence and is influenced by some psychopathological aspects that are not strictly correlated to the eating symptomatology as interoceptive deficits mainly and partially as the emotional dysregulation and the overcontrol. Differently, in females BMI associate with drive for thinness and with the risk of develop an ED, aspects more strictly correlate to the EDs symptomatology. These results, in line with results of Gila and collaborators [38] on a sample of adolescents with anorexia nervosa, may suggest that boys appear to worry less about weight as they progress through adolescence, the reverse of the process characteristically seen in adolescent girls. The authors hypothesize that these results suggest that body development is evaluated differently by boys and girls: whereas in boys the pubertal increase of body mass is to a large extent desirable, in girls it can be perceived as a threat. These aspects, if confirmed in further research, may constitute a peculiar and gendered-based characteristics of adolescent males with EDs.

## Strength and limits

Limitations to this study include the sample size and the exclusive use of self-report to assess the psychological variables. The enlargement of the males’ size and more rigorous approaches to the measurement, for instance by combining self-report instruments with interview measures or the adoption of gender-specific rating scales would be important. Conversely, the strengths of this study include a homogeneous and well-characterized sample of adolescents, improving the quality of the results and reducing possible bias.

## Conclusion

In conclusion, our research confirms that EDs in adolescence show different peculiarities and some similarities in males in comparison to females. In fact, if adolescent males and females show similar profiles in term of diagnosis and BMI and eating symptomatology in general, adolescent males show a peculiar and more severe psychopathological profiles partially influenced by more severe BMI and characterized by purging-behaviours, over-exercising, obsessive–compulsive behaviour, anxiety, and psychoticism. In other words, our results suggest that EDs in adolescent males seem to be supported by a different impairment of psychic functioning than females, in which the problems linked to the body and the eating behaviours seem to be the tip of the iceberg of very different clinical and temperamental characteristics and in which the symptoms related to the specific fear of gaining weight and the impulse to lose weight are less predominant. Although studies involving larger samples of adolescent males are still needed, likewise the definition of gender-specific diagnostic tools, this study gives an important contribution to understand and analyse the psychological profile of this population, confirming the hypothesis that adolescent males show different clinical expression of EDs in comparison to females. Clinical implications of these results include the necessity to define specific and gendered treatment programmes, oriented to the resolution not only of the eating core symptomatology, but also to the comorbidity, considering their strictly correlations with the BMI. Another implication of our results includes the need to increase the awareness of these topics, and to implement prevention strategies in populations at risk (e.g., athletes).

## References

[1] Herpertz-Dahlmann B (2015). Adolescent eating disorders: update on definitions, symptomatology, epidemiology, and comorbidity. Child Adolesc Psychiatr Clin N Am.

[2] Shisslak CM, Crago M, Estes LS (1995). The spectrum of eating disturbances. Int J Eat Disord.

[3] APA American Psychiatric Association (2013). Diagnostic and statistical manual of mental disorders.

[4] Smink FRE, Van Hoeken D, Hoek HW (2013). Epidemiology, course, and outcome of eating disorders. Curr Opin Psychiatry.

[5] Mancini G, Biolcati R, Pupi V (2018). I disturbi della nutrizione e dell’alimentazione nei maschi: Una panoramica sulle ricerche nel periodo 2007–2017. Riv Psichiatr.

[6] Murray SB, Nagata JM, Griffiths S (2017). The enigma of male eating disorders: a critical review and synthesis. Clin Psychol Rev.

[7] Manzato E (2019). Eating disorders and disordered eating behaviors in males: a challenging topic. Eat Weight Disord.

[8] Dearden A, Mulgrew KE (2013). Service provision for men with eating issues in Australia: an analysis of organisations’, practitioners’, and men’s experiences. Aust Soc Work.

[9] Murray SB, Griffiths S, Mond JM (2016). Evolving eating disorder psychopathology: conceptualising muscularity-oriented disordered eating. Br J Psychiatry.

[10] Raevuori A, Keski-Rahkonen A, Hoek HW (2014). A review of eating disorders in males. Curr Opin Psychiatry.

[11] Quadflieg N, Naab S, Voderholzer U, Fichter MM (2022). Long-term outcome in males with anorexia nervosa: a prospective, sex-matched study. Int J Eat Disord.

[12] Nagata JM, Ganson KT, Murray SB (2020). Eating disorders in adolescent boys and young men: an update. Curr Opin Pediatry.

[13] Gorrell S, Murray SB (2019). Eating disorders in males. Child Adolesc Psychiatr Clin N Am.

[14] Mitchison D, Mond J (2015). Epidemiology of eating disorders, eating disordered behaviour, and body image disturbance in males: a narrative review. J Eat Disord.

[15] Limbers CA, Cohen LA, Gray BA (2018). Eating disorders in adolescent and young adult males: prevalence, diagnosis, and treatment strategies. Adolesc Health Med Ther.

[16] Shingleton R, Thompson-brenner H, Thompson DR, Pratt EM, Franko DL (2015). Gender differences in clinical trials of binge eating disorder: an analysis of aggregated data. J Consult Clin Psychol.

[17] Tylka TL, Subich LM (2002). A preliminary investigation of the eating disorder continuum with men. J Couns Psychol.

[18] McCreary DR, Sasse DK (2000). An exploration of the drive for muscularity in adolescent boys and girls. J Am Coll Health Assoc.

[19] Chen DR, Sun G, Levin B (2022). Gender-specific responses to multifaceted factors associated with disordered eating among adolescents of 7th to 9th grade. J Eat Disord.

[20] Button E, Aldridge S, Palmer R (2008). Males assessed by a specialized adult eating disorders service: patterns over time and comparisons with females. Int J Eat Disord.

[21] Strother E, Lemberg R, Stanford SC, Turberville D (2012). Eating disorders in men: underdiagnosed, undertreated, and misunderstood. Eat Disord.

[22] Lewinsohn PM, Seeley JR, Moerk KC, Striegel-Moore RH (2002). Gender differences in eating disorder symptoms in young adults. Int J Eat Disord.

[23] Anderson CB, Bulik CM (2004). Gender differences in compensatory behaviors, weight and shape salience, and drive for thinness. Eat Behav.

[24] Gordon AR, Moore LB, Guss C, Nagata JM, Brown TA, Murray SB, Lavender JM (2021). Eating disorders among transgender and gender non-binary people. Eating disorders in boys and men.

[25] Ulfvebrand S, Birgegård A, Norring C, Högdahl L, von Hausswolff-Juhlin Y (2015). Psychiatric comorbidity in women and men with eating disorders results from a large clinical database. Psychiatry Res.

[26] Grilo CM, White MA, Masheb RM (2009). DSM-IV psychiatric disorder comorbidity and its correlates in binge eating disorder. Int J Eat Disord.

[27] Valente S, Di Girolamo G, Forlani M (2017). Sex-specific issues in eating disorders: a clinical and psychopathological investigation. Eat Weight Disord.

[28] Claes L, Jiménez-Murcia S, Agüera Z (2012). Male eating disorder patients with and without non-suicidal self-injury: a comparison of psychopathological and personality features. Eur Eat Disord Rev.

[29] Nagata JM, Bojorquez-Ramirez P, Nguyen A (2022). Sex differences in refeeding among hospitalized adolescents and young adults with eating disorders. Int J Eat Disord.

[30] Ridout SJ, Ridout KK, Kole J, Fitzgerald KL, Donaldson AA, Alverson B (2021). Comparison of eating disorder characteristics and depression comorbidity in adolescent males and females: an observational study. Psychiatry Res.

[31] Riva A, Pigni M, Albanese ND (2022). Eating disorders in children and adolescent males: a peculiar psychopathological profile. Int J Environ Res Public Health.

[32] Giannini M, Pannocchia L, Dalle Grave R, Muratori F (2008) Italian version of the eating disorder inventory-3. Edizioni O. S. Giunti

[33] Clausen L, Rosenvinge JH, Friborg O, Rokkedal K (2011). Validating the eating disorder inventory-3 (EDI-3): a comparison between 561 female eating disorders patients and 878 females from the general population. J Psychopathol Behav Assess.

[34] Prunas A, Sarno I, Preti E, Madeddu F, Perugini M (2012). Psychometric properties of the Italian version of the SCL-90-R: a study on a large community sample. Eur Psychiatry.

[35] Shaffer D, Gould MS, Brasic J, et al (1983) A Children’s Global Assessment Scale. pp 5–8 10.1001/archpsyc.1983.01790100074010 6639293

[36] Downey CA, Reinking KR, Gibson JM, Cloud JA, Chang EC (2014). Perfectionistic cognitions and eating disturbance: distinct mediational models for males and females. Eat Behav.

[37] Murray SB, Pila E, Mond JM (2018). Cheat meals: a benign or ominous variant of binge eating behavior?. Appetite.

[38] Gila A, Castro J, Cesena J, Toro J (2005). Anorexia nervosa in male adolescents: body image, eating attitudes and psychological traits. J Adolesc Heal.

[39] Halmi KA, Tozzi F, Thornton LM (2005). The relation among perfectionism, obsessive–compulsive personality disorder and obsessive–compulsive disorder in individuals with eating disorders. Int J Eat Disord.

[40] Jiménez-Murcia S, Fernández-Aranda F, Raich RM (2007). Obsessive-compulsive and eating disorders: comparison of clinical and personality features. Psychiatry Clin Neurosci.

[41] Bean P, Maddocks MB, Timmel P, Weltzin T (2005). Gender differences in the progression of co-morbid psychopathology symptoms of eating disordered patients. Eat Weight Disord.

[42] Weltzin T, Cornella-Carlson T, Weisensel N, Timmel P, Hallinan P, Bean P (2007). The combined presence of obsessive-compulsive behaviors in males and females with eating disorders account for longer lengths of stay and more severe eating disorder symptoms. Eat Weight Disord.

[43] Carlat DJ, Camargo CA, Herzog DB (1997). Eating disorders in males: a report on 135 patients. Am J Psychiatry.

[44] Tanofsky MB, Wilfley DE, Spurrell EB, Welch R, Brownell KD (1997). Comparison of men and women with binge eating disorder. Int J Eat Disord.

[45] Koyanagi A, Stickley A, Haro JM (2016). Psychotic-like experiences and disordered eating in the English general population. Psychiatry Res.

